# A Novel F-Box Protein CaF-Box Is Involved in Responses to Plant Hormones and Abiotic Stress in Pepper (*Capsicum annuum* L.)

**DOI:** 10.3390/ijms15022413

**Published:** 2014-02-10

**Authors:** Rugang Chen, Weili Guo, Yanxu Yin, Zhen-Hui Gong

**Affiliations:** 1 College of Horticulture, Northwest A&F University, Yangling 712100, Shaanxi, China; E-Mails: rugangchen@126.com (R.C.); guoweili1226@sina.com (W.G.); 2 State Key Laboratory of Crop Stress Biology in Arid Areas, Northwest A&F University, Yangling 712100, Shaanxi, China; E-Mail: yinyanxu2008@nwsuaf.edu.cn (Y.Y); 3 School of Horticulture Landscape Architecture, Henan Institute of Science and Technology, Xinxiang 453003, Henan, China

**Keywords:** *Capsicum annuum*, F-box protein, abiotic stress response, plant hormones, expression analysis, gene silencing

## Abstract

The F-box protein family is characterized by an F-box motif that has been shown to play an important role in regulating various developmental processes and stress responses. In this study, a novel F-box-containing gene was isolated from leaves of pepper cultivar P70 (*Capsicum annuum* L.) and designated *CaF-box*. The full-length cDNA is 2088 bp and contains an open reading frame of 1914 bp encoding a putative polypeptide of 638 amino acids with a mass of 67.8 kDa. *CaF-box* was expressed predominantly in stems and seeds, and the transcript was markedly upregulated in response to cold stress, abscisic acid (ABA) and salicylic acid (SA) treatment, and downregulated under osmotic and heavy metal stress. *CaF-box* expression was dramatically affected by salt stress, and was rapidly increased for the first hour, then sharply decreased thereafter. In order to further assess the role of *CaF-box* in the defense response to abiotic stress, a loss-of-function experiment in pepper plants was performed using a virus-induced gene silencing (VIGS) technique. Measurement of thiobarbituric acid reactive substances (TBARS) and electrolyte leakage revealed stronger lipid peroxidation and cell death in the *CaF-box*-silenced plants than in control plants, suggesting *CaF-box* plays an important role in regulating the defense response to abiotic stress resistance in pepper plants.

## 1. Introduction

The development and functioning of an organism requires exquisite coordination of the cellular responses to internal and external signals. One important mechanism by which this is achieved is through control of the structural and functional integrity of the numerous key regulators using protein degradation by the ubiquitin proteosome system (UPS) [1]. Regulation of protein degradation by the UPS is a highly conserved process and plays an important role in cellular processes as diverse as differentiation, cell cycle regulation, hormonal responses, protein trafficking, and response to environmental stress [1–3]. The ubiquitin-conjugation pathway involves the sequential action of three enzymes or protein complexes: E1 (ubiquitin-activating enzyme), E2 (ubiquitin-conjugating enzyme), and E3 (ubiquitin-protein ligase). In most cases, E1 and E2 are relatively nonspecific and are responsible for activating and conjugating the ubiquitin moieties, whereas different E3 enzymes recognize different substrates for ubiquitination [4]. One of the best characterized and the most important E3 type ligases are the SCF (Skp–Cullin–F-box) protein complexes formed from four major components: Skp1, Cullin, Rbx1, and an F-box protein [1,5]. In the SCF complex, the first three proteins form a core scaffold onto which different F-box proteins can be assembled, conferring specificity to the complex [6].

F-box proteins are encoded by a rapidly expanding family of eukaryotic genes. They consist of a conserved domain of 40–60 residues at the N-terminus known as the F-box that was first identified in cyclin F and mediates the interaction with Skp1. Many F-box proteins have one or several highly variable protein-protein interaction domains at the *C*-terminus, such as Leu-rich repeats (LRRs), WD40 repeats, tertratricopeptide repeats (TPR), or kelch repeats, and these are thought to confer the substrate specificity for ubiquitination [7,8]. Since the discovery of the first F-box protein (Cyclin F) in human, many F-box proteins have been identified in a host of different organisms. A total of 14, 24, 337, and 38 *F-box* genes have been reported in budding yeast, fruit fly, nematode, and human, respectively [9]. In plants, more than 692, 337, and 779 *F-box* genes have been reported in *Arabidopsis*, poplar, and rice genomes, respectively [10].

Several *F-box* genes have been characterized which regulate crucially important and diverse physiological processes, such as hormonal response, embryogenesis, seed germination, seedling development, floral organogenesis, lateral root formation, leaf senescence, pathogen resistance, and abiotic stress responses [11–13]. UNUSUAL FLORAL ORGANS (UFO) was the first F-box protein identified in *Arabidopsis*, and UFO is required for floral meristem identity and floral organ development [14,15]. TIR1 and COI1 include LRR domains, and are responsible for auxin and jasmonic acid (JA) signal transduction, respectively [16,17]. The ORE9 protein regulates leaf senescence [18], while SON1 and CPR30 play key roles as negative regulators in plant defense responses to pathogens [19,20]. In addition, it have been reported that the rice F-box protein OsFbx352 played a key regulatory role in the regulation of glucose-induced suppression of seed germination by targeting ABA metabolism [13]. Zhang *et al.* (2008) found that the F-box protein DOR acts as a negative regulator during response to ABA and drought stress [21]. Another F-box protein, CarF-box1, is involved in various plant developmental processes and abiotic stress responses [22]. Yan *et al.* (2011) reported that overexpression of the rice F-box protein *MAIF1* reduced abiotic stress tolerance and promoted root growth, and a negative role in response to abiotic stress was suggested, possibly through regulating root growth [23].

To date, no *F-box* genes have been characterized in pepper (*Capsicum annuum* L.). In this study, we isolated *CaF-box* from the leaves of pepper cultivar P70. *CaF-box* showed tissue-specific expression in different organs of pepper plants. To investigate possible roles in the defense response against abiotic stresses (cold, salt, osmotic, and heavy metal stress), as well as in plant hormone signaling (abscisic acid and salicylic acid), the expression of *CaF-box* was analyzed using Real-Time quantitative RT-PCR. Furthermore, *CaF-box* loss-of-function mutant pepper plants were engineered using a virus-induced gene silencing (VIGS) system. Together, the results suggested that *CaF-box* is a potentially important player in the regulation of plant defense responses.

## 2. Results and Discussion

### 2.1. Isolation of CaF-Box and Sequence Analysis

**Figure 1.**
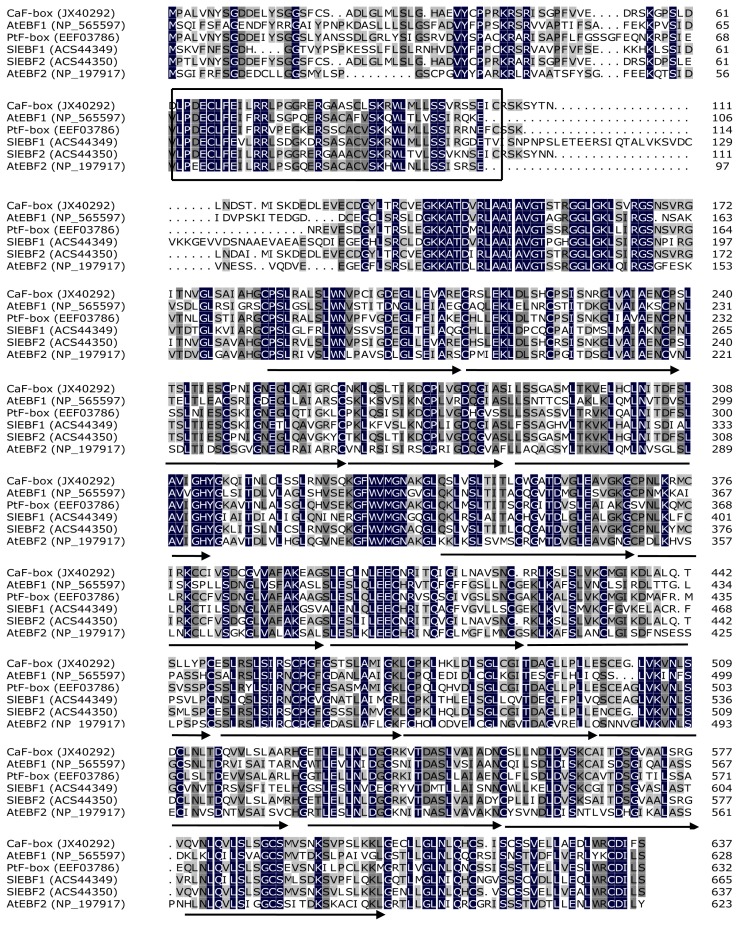
Multiple sequence alignment of CaF-box with the amino acid sequences of *Arabidopsis thaliana* AtEBF1 and AtEBF2, *Solanum lycopersicum* SlEBF1 and SlEBF2, and *Populus trichocarpa* PtF-box. Putative F-box motif sequences are boxed, and the 15 deduced leucine-rich repeats (LRRs) are indicated by arrows under the sequences.

A pepper cDNA clone was selected from the differential screening of the cold-related pepper seeding cDNA library, which had been prepared by PCR-amplified subtracted and control probes [24]. This clone exhibited 90% sequence identity at the nucleotide level with *SlEBF2*, an *F-box* protein-encoding gene from *Solanum lycopersicum*. Based on the sequence of the fragment, 3′- and 5′-RACE primers were designed and RACE was performed which generated two fragments of 1325 and 984 bp, respectively. Through alignment and assembly of these three sequences, the full-length cDNA was deduced, amplified by PCR, and confirmed by sequencing. The 2088 bp full-length cDNA contains a 1914 bp ORF and encodes a 638 amino acid polypeptide with a calculated isoelectric point of 6.96 and theoretical molecular mass of 67.8 kDa. This gene was designated as *CaF-box* and submitted to GenBank with accession number JX402925. An NCBI blast search indicated that the deduced CaF-box amino acid sequence showed moderate homology with other F-box proteins from *Solanum lycopersicum* (SlEBF1, GenBank accession no. ACS44394, 55.2% identity, and SlEBF2, GenBank accession no. ACS44350, 85.2% identity), *Arabidopsis thaliana* (AtEBF1, GenBank accession no. NP_565597, 51.2% identity, and AtEBF2, GenBank accession no. NP_197917, 50.3% identity), and *Populus trichocarpa* (PtF-box, GenBank accession no EEF03786, 66.0% identity). Two characteristic protein domains were identified in the CaF-box sequence: an F-box domain (residues 63–104) in the *N*-terminal region, and 15 cascade Leu-rich repeats (LRRs) in the *C*-terminal region (Figure 1).

**Figure 2.**
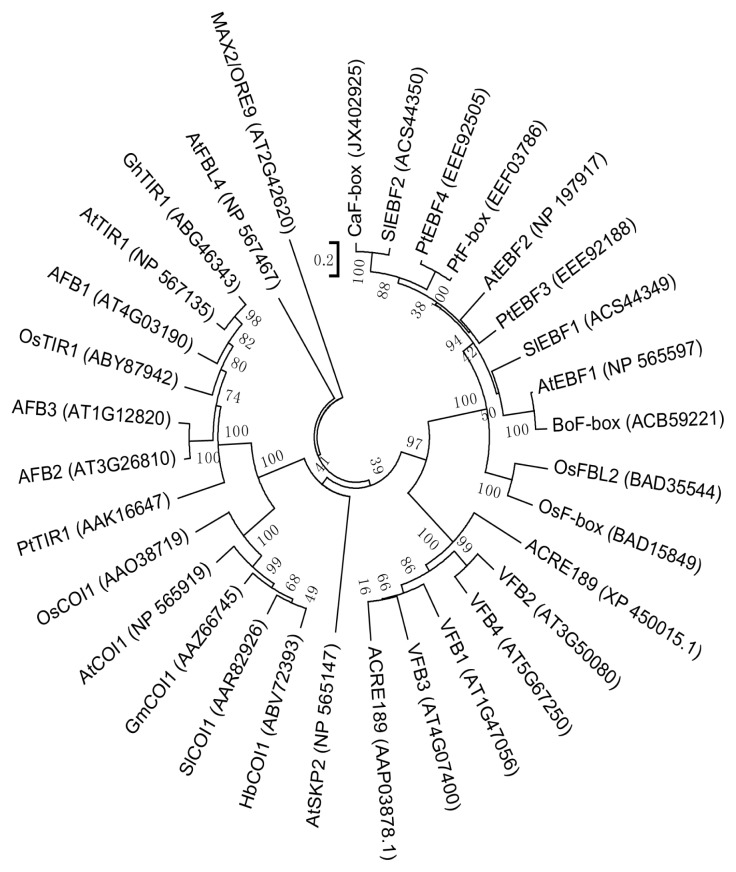
Phylogenetic tree of CaF-box and homologous F-box proteins from other species. The rooted gene tree (majority-rule consensus from 1000 bootstrap replicates) was constructed using the heuristic searching option in Mega5.1. Bootstrap values are indicated at each branch node. GenBank accession numbers are in parentheses after each species and gene name. The scale bar shows the similarity coefficient.

In order to evaluate the molecular evolutionary relationships between CaF-box and other F-box proteins, a phylogenetic tree was prepared using Mega5.1 (Figure 2). CaF-box fell into the EBF-type F-box protein family, and the bioinformatics analysis suggested CaF-box is most highly related to plant F-box proteins that are involved in formation of E3 ubiquitin ligase complexes.

### 2.2. Expression Patterns of CaF-Box in Different Tissues of Pepper Plants

**Figure 3.**
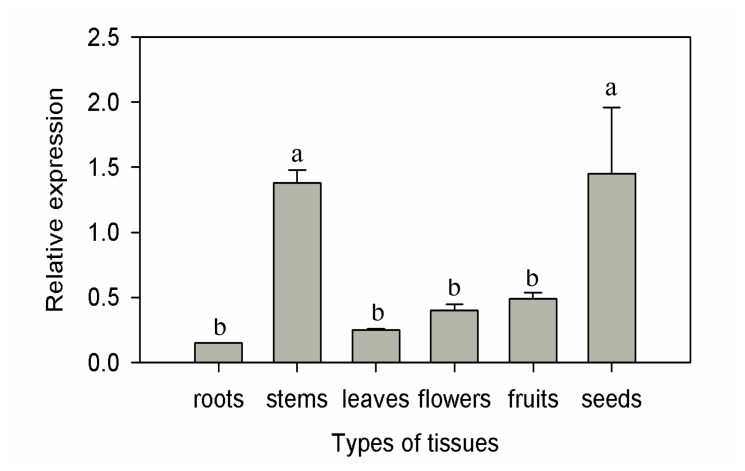
Tissue-specific expression of *CaF-box* in different tissues of pepper plants. Error bars represent the mean ± SD of three independent biological replicates. Different letters indicate significant differences (*p <* 0.05).

Analysis of gene expression patterns provides useful information for speculation concerning protein function [22]. To investigate the physiological role of CaF-box, the basal expression in different pepper tissues including roots, leaves, stems, flowers, fruits, and seeds were analyzed by real-time quantitative PCR (Figure 3). *CaF-box* was expressed ubiquitously in all tissues, and the basal expression was similar in roots, leaves, flowers, and fruits, but much higher in stems and seeds. The result is in agreement with previous studies on the expression patterns of *F-box* protein-encoding genes that showed basically expressed in all organs [25,26]. The higher expression levels in stems and seeds suggest that *CaF-box* gene may play a role in development.

### 2.3. Expression Patterns of *CaF-Box* Gene in Response to Abiotic Stress and Plant Hormones Treatment

Plants have evolved several elaborate regulatory defense strategies to protect themselves from various biotic and abiotic stresses [27]. To investigate the relationship between pepper *CaF-box* and stress responses, the transcription level of *CaF-box* under abiotic stress conditions (cold, high salinity, osmotic, and heavy metal stress) was measured by real-time quantitative PCR.

#### 2.3.1. Expression Analysis of *CaF-Box* Gene in Response to Cold, Salt, Osmotic, and Heavy Metal Stress Treatments

**Figure 4.**
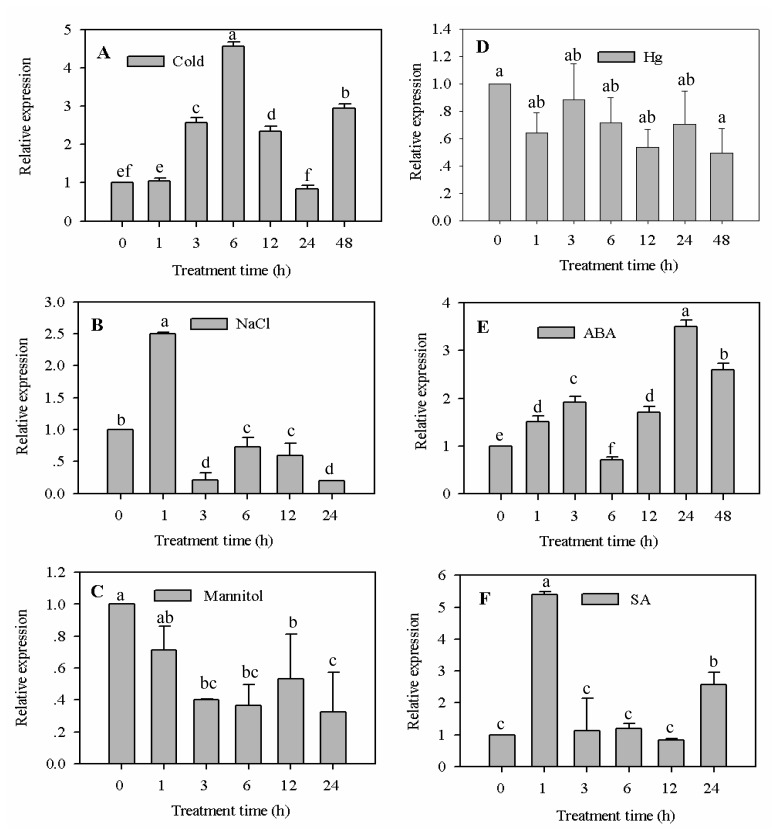
Real-time RT-PCR analysis of relative *CaF-box* expression levels in the leaves of pepper plants after abiotic stress and plant hormone treatments. (**A**) Cold stress; (**B**) Salt stress; (**C**) Osmotic stress; (**D**) Heavy metal stress; (**E**) ABA treatment; (**F**) SA treatment. Error bars represent the mean ± SD of three independent biological replicates. Different letters indicate significant differences (*p <* 0.05).

F-box proteins are highly abundant in plants, and many are involved in the regulation of plant defense responses [21,28]. In this study, *CaF-box* exhibited a diverse expression pattern in response to cold, salt, osmotic, and heavy metal stresses (Figure 4).

Temperature stress is one of the most important abiotic factors limiting the growth, development and geographical distribution of plants [29], and the expression of many defense genes are altered in response to low or high temperature stress. In this study, *CaF-box* was markedly upregulated by cold stress (Figure 4A). Increased levels of *CaF-box* transcript were detectable within 3 h after cold treatment, with the maximal level reached at 6 h after treatment. However, *CaF-box* transcription was downregulated at 24 h, but then upregulated again at 48 h after treatment. The transcript level of *CaF-box* gene was similar to the EST R029 transcript in pepper seedlings subjected to cold stress [24]. And this was reminiscent of the findings of Yan *et al.* (2011) who found that low temperature rapidly and strongly induced the expression level of *MAIF1*, an *F-box* protein-encoding gene from rice [23]. In contrast, the *F-box* gene *CarF-box* from chickpea was downregulated by cold stress [22]. Together, these results indicate that different *F-box* genes exhibit different expression patters under cold stress conditions.

In contrast to the results for cold stress, *CaF-box* expression was significantly enhanced in pepper leaves under salt treatment, with a 2.5-fold increase 1 h after a 300 mM NaCl treatment (Figure 4B). However, *CaF-box* was dramatically downregulated 3 h after NaCl treatment, with mRNA levels only 0.2 fold that of control plants. This level of downregulation was also measured 24 h after salt stress. These expression patterns were similar to previous observations [30]. Expression of the *Arabidopsis F-box* protein-encoding gene of *FOA1* was rapidly induced following NaCl treatment, with levels peaking at 2 h and then gradually decreasing with time. These results suggested that *CaF-box* could be involved in responses to salt stress in pepper.

Previous studies demonstrated that osmotic and heavy metal stress induced a wide range of different changes in gene expression and/or physiological responses [31,32]. In this study, expression f *CaF-box* was downregulated in response to both osmotic and heavy metal stress (Figure 4C,D), suggesting *CaF-box* may play a negative role in response to these stresses.

#### 2.3.2. Expression Analysis of *CaF-Box* Gene in Response to ABA and SA Treatment

Plant hormones such as ABA and SA are important signaling molecules that contribute to biotic and abiotic stress tolerance in pepper [33–35]. To investigate a possible involvement of CaF-box in defense-associated signaling, expression was analyzed in pepper leaves treated with 0.57 mM ABA or 5 mM SA using real-time quantitative PCR.

ABA treatment dramatically upregulated CaF-box expression (Figure 4E). At 3 h after treatment, CaF-box transcription was elevated two-fold compared to control plants. However, there was a sharply decrease in CaF-box mRNA levels at 6 h as transcript levels reached their lowest, before gradually increasing, and transcription levels peaked 24 h after treatment. This pattern was consistent with previous studies in which ABA enhanced the expression of *F-box* genes. Gao *et al.* (2010) found that exogenous application of ABA caused a rapid induction in expression of *BnSLY1*, an F-box protein-encoding gene in *Brassica napus* [36]. Transcript levels of OsFox352 also rapidly accumulated following ABA treatment [13]. These results suggested that CaF-box may be involved in the ABA-dependent signaling pathway.

Following treatment with 5 mM SA, *CaF-box* transcript levels were strongly induced 5.5-fold compared with control plants after 1 h, before levels fell sharply and were maintained at low levels between 3 and 12 h (Figure 4F). At 24 h, expression levels were rapidly increased by 2.8-fold. Similarly Paquis *et al.* (2011) also reported that exogenous application of SA strongly induced the expression of *BIG-24.1*, an *F-box* protein-encoding gene in grapevine (26). The results showed that exogenous SA rapidly induced the transcription of *CaF-box*, suggesting an involvement in SA signaling.

Taken together, these results indicate that *CaF-box* is involved in various abiotic stresses, as well as ABA- and SA-dependent signal transduction pathways. This makes *CaF-box* a potentially valuable gene for future genetic engineering of plants for enhanced abiotic stress tolerance.

### 2.4. Suppression of *CaF-Box* Results in Reduced Tolerance to Cold Stress

**Figure 5.**
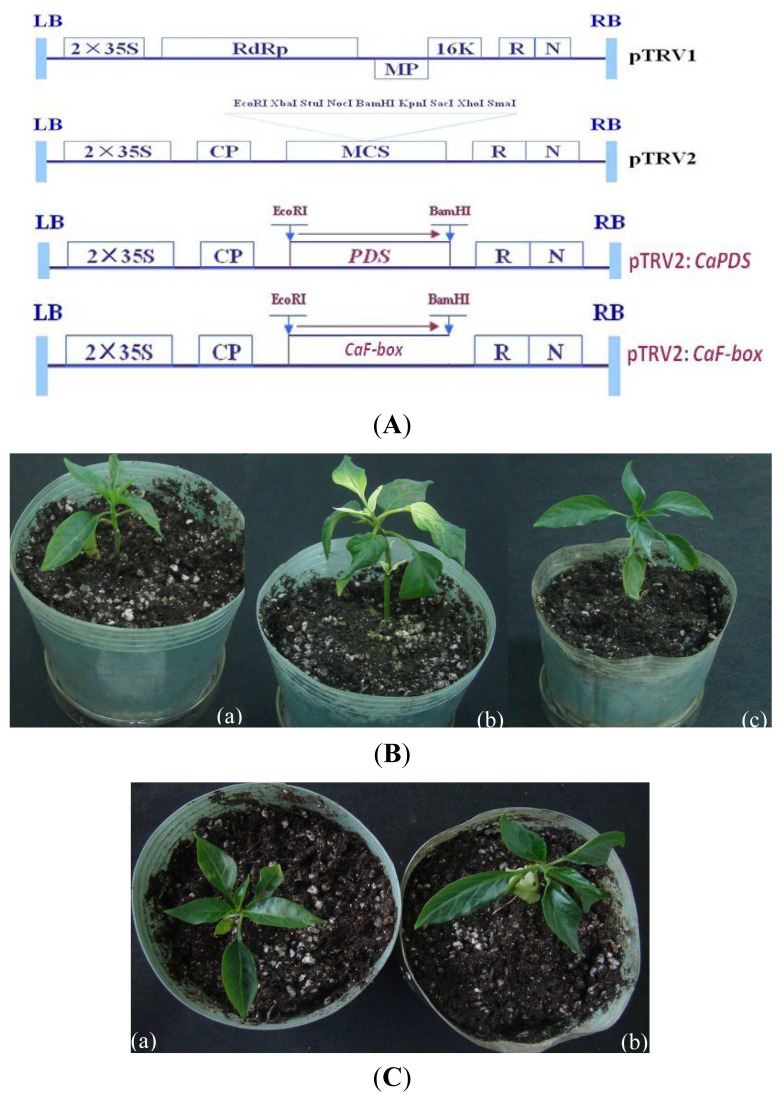

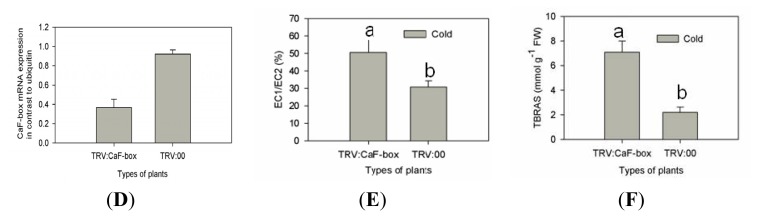
VIGS of *CaF-box* gene in the pepper plant. (**A**) Schematic representation of the tobacco rattle virus (TRV), pTRV2:*CaPDS* and pTRV2:*CaF-box* constructs; (**B**) Phenotypes of gene silencing pepper plants. (**a**) Control plant (pTRV2-00); (**b**) *CaPDS*-silenced plant (pTRV2:*CaPDS*); (**c**) *CaF-box-*silenced plant (pTRV2:*CaF-box*); (**C**) Phenotypes of the control plant and gene silenced plant after 24 h 6 °C treatment. (**a**) Control plant (pTRV2-00); (**b**) *CaF-box-*silenced plant (pTRV2:*CaF-box*); (**D**) Real-time RT-PCR analysis of relative *CaF-box* expression levels in gene-silenced (pTRV:*CaF-box*) and control (pTRV:00) plants 45 days after inoculation; (**E**) Effects of low temperature stress on EC1/EC2; and (**F**) TBARS in *CaF-box-*silenced pepper seedlings. Asterisks indicate significant differences compared to empty vector control leaves based on the least significant difference (LSD) test (*p* < 0.05). Different letters indicate significant differences (*p <* 0.05).

To examine further the role of CaF-box in the pepper plant cold stress defense response, virus-induced gene silencing (VIGS) was performed in pepper cultivar P70 using the tobacco rattle virus (TRV)-base virus-induced gene silencing technique [37,38]. A fragment from the 3′ end of the *CaF-box* ORF was cloned into the pTRV2 vector to generate the pTRV2:*CaF-box* construct. Empty vector (pTRV:00) was used as a negative control. The pTRV2-*CaPDS*, which silences phytoene desaturase gene (*PDS*) and induces a photo-bleaching phenotype, was used as a positive control to determine the success of gene silencing (Figure 5A).

As shown in Figure 5B, 35 days after the induction of TRV-mediated gene silencing, pepper plants that have been infiltrated with pTRV2:CaF-box showed no visible phenotype when compared to negative control plants (Figure 5B), but the symptoms of photo-bleaching were occurred on the leaves of *CaPDS* silenced plants. To verify that the *CaF-box* transcript was effectively down-regulated by VIGS, real-time quantitative PCR was carried out (Figure 5D). *CaF-box* expression was dramatically reduced by 60% in new leaves between the silenced and pTRV:00 infiltrated pepper plants grown at 22 °C under non-stress conditions, suggesting that VIGS was successful and effective for *CaF-box* gene silencing in pepper.

Under cold stress, the pTRV:CaF-box plants aggravated the visible symptoms of leaf damage in seedlings. There was more seriously wilting appeared in pepper seedlings in pTRV:CaF-box plants than that of pTRV:00 plants after 24 h 6 °C treatment (Figure 5C). In order to further confirm the influence of silencing of *CaF-box* in the cold stress defense response, the TBARS and electrical conductivity measurements in pepper leaves was tested in the control plant (pTRV-00) and *CaF-box* silenced plant (pTRV2-*CaF-box*). As shown in Figure 5E,F, after 24 h of 6 °C cold treatments, the TBARS and electrical conductivity measurements were significantly increased in *CaF-box*-silenced plants compared to control plants. Numerous studies have reported that cold stress induces dehydration, which eventually leads to wilting and results in membrane disintegration and electrolyte leakage [39]. TBARS and electrical conductivity measurements are two established indicators of the extent of cell membrane injury and electrolyte leakage, and are known to correlate well with the severity of the visual damage index [40]. In this study, cold stress significantly increased both TBARS and electrical conductivity in the *CaF-box*-silenced plants, suggesting loss of *CaF-box* reduced the tolerance of pepper seedlings to cold treatment, indicating a potential role for *CaF-box* in resistance to cold stress.

**Figure 6.**
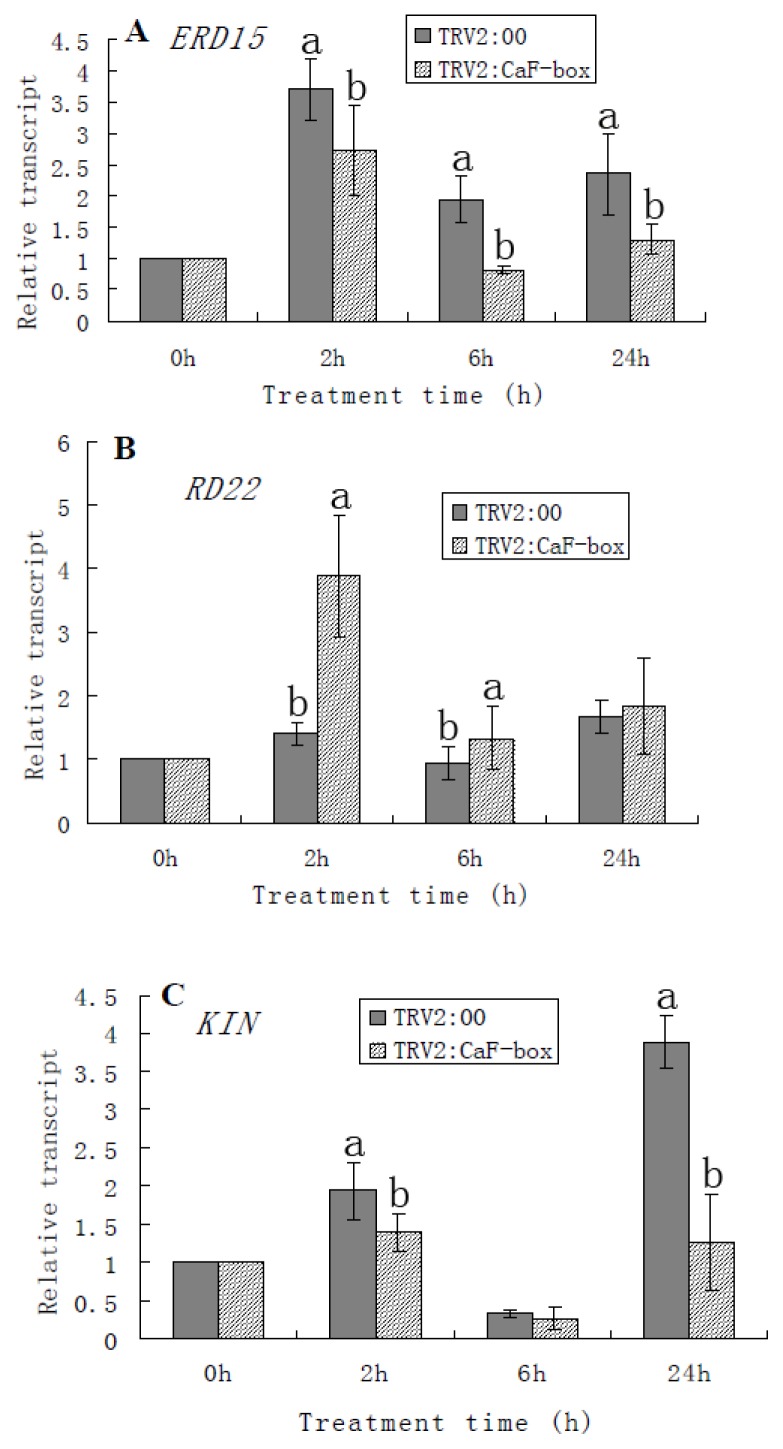
Expression of cold stress-responsive genes in control and *CaF-box* silenced plants subjected to cold stress. Relative expression levels of cold stress-responsive genes were determined by real time PCR. (**A**) *ERD15* gene; (**B**) *RD22* gene; (**C**) *KIN1* gene. Three biological triplicates were averaged and Bars indicate standard error of the mean. Different letters indicate significant differences (*p <* 0.05).

To elucidate the mechanism of reduced tolerance to cold stress in *CaF-box*-silenced plants, the expression patterns of several cold-related genes were monitored in both negative control (pTRV-00) and *CaF-box-*silenced plants (pTRV2-*CaF-box*) by real-time quantitative PCR (Figure 6).

Earlier studies have found *ERD15*, *RD22*, and *KIN1* to be involved in the response to dehydration and cold stress [41–43]. Compared with normal conditions, cold stress induced *ERD15*, *RD22*, and *KIN1* (except at 6 h) gene expression in both control plant (pTRV-00) and *CaF-box-*silenced plant (pTRV2-*CaF-box*) (Figure 6A–C). After cold treatment, the expressions of *ERD15* and *KIN1* in *CaF-box-*silenced plants were lower than that in the control plants (Figure 6A,C). However, the result of *RD22* transcript was reversed (Figure 7B). These results indicated that knock down the *CaF-box* gene suppressed chilling-induced *ERD15* and *KIN1* transcripts. Therefore, *CaF-box* appeared to act as a positive regulator of cold stress-responsive gene expression, such as *ERD15* and *KIN1*, consistent with the results from leaf chilling injury assays.

## 3. Experimental Section

### 3.1. Plant Materials and Growth Conditions

Pepper cultivar P70 was provided by the pepper breeding group in Northwest A&F University, China. Seeds were soaked in warm water (55 °C) for 20 min to promote germination, rinsed twice per day, and placed on moist gauze in an incubator at 28 °C, 60% relative humidity in darkness. When seeds were at least 80% germinated, they were sown in a soil mix of 3:1 grass charcoal:perlite (*v*/*v*) in plastic pots. Pepper plants were grown in a growth chamber with a 16 h light and 8 h dark photoperiod at 25 °C.

### 3.2. Bacterial Strains, Vectors, Enzymes

Vector pMD19-T (TaKaRa, Dalian, China) and pTRV2 were used to engineer constructs, and *Agrobacterium tumefaciens* strain GV3101 was used for subsequent plant transformation. *Pfu* DNA polymerase (Promega Corporation, Madison, WI, USA) was used for PCR, and products were subsequently sequenced. T4 DNA ligase (TaKaRa, Dalian, China), *Bam*HI and *Xba*I restriction enzymes (Promega Corporation, Madison, WI, USA) were used for vector construction.

### 3.3. Isolation of *CaF-Box* cDNA Clone and Sequence Analysis

The *F-box*-homologous EST R029 (GenBank No: JZ198812) characterized from the differential screening of a cold-related pepper seedling cDNA library was previously reported [24]. The full-length *CaF-box* ORF was obtained using the rapid amplification of cDNA ends (RACE) method. First-strand cDNA synthesis was performed using the Smart RACE cDNA amplification kit (Clontech, Mountain View, CA, USA). Gene-specific primer GSP1 (5′-GTCTTGTCGCAATAGCAG-3′) was used for 3′-RACE and GSP2 (5′-GTTTAGACAGTGTAGTTCCA-3′) was used for 5′-RACE. Universal primers for 5′ and 3′ RACE were provided in the kit. The full-length cDNA sequence of *CaF-box* was obtained by PCR amplification using forward (5′-CTTGGTTGTGATTTTCTTGG-3′) and reverse (5′-CACTCGTGTTTGCTTCTGTA-3′) primers. PCR products were cloned into the pMD19-T vector (Takara) and sequenced (Shanghai GeneCore Biotechnologies Co., Shanghai, China).

NCBI bioinformatics tools were used to analyze nucleotide and protein sequence data. Conserved protein domains were identified using the Conserved Domain database, and the theoretical molecular weight (*M*_w_) and isoelectric point (pI) were calculated using the ExPASy compute pI/*M*_w_ tool [44]. Multiple sequence alignments of *CaF-box* and F-box proteins from other species were performed using Clustal W [45]. The phylogenetic tree was constructed using Mega5.1 by the neighbor-joining method.

### 3.4. *CaF-Box* Gene Expression Patterns Analysis

#### 3.4.1. Tissue-Specific Expression of *CaF-Box* Gene

To evaluate the expression levels of *CaF-box* in different tissues under normal conditions, roots, stems, leaves, flowers, fruits and seeds were collected from pepper cultivar P70 plants, frozen in liquid nitrogen, and stored at −80 °C until needed for gene expression analysis.

#### 3.4.2. Stress Treatments

Pepper seedlings at the sixth leaf expansion stage were used to establish abiotic stress and plant hormone treatments. ABA and cold treatments were performed as described previously [39]. For salt, osmotic, and heavy metal (Hg) treatments, seedling roots were immersed in solutions containing 300 mM sodium chloride (NaCl), 300 mM mannitol, or 50 μM Hg, and maintained at 25 °C for the indicated times. For SA treatment, seedlings were sprayed with 5 mM SA solution and incubated for the indicated times. Treated seedlings were harvested after 0, 1, 3, 6, 12, and 24 h for examination of *CaF-box* expression under stress conditions. At each time point, two or three upper young leaves from four separate seedlings were collected and combined to form one sample, wrapped in aluminium foil, immediately frozen in liquid nitrogen and stored at −80 °C. The treatments were arranged in a randomized complete block design and included three experiments. Each experiment includes three replicates.

### 3.5. RNA Isolation and Real-Time RT-PCR Analysis

Total RNA was extracted from pepper leaves at different time points after diverse stress treatments using the Trizol (Invitrogen, Grand Island, NY, USA) method. RNA concentration was measured spectrophotometrically using a NanoDrop 2000C instrument (Thermo Scientific, Pittsburgh, PA, USA), and the purity was assessed using the A260/280 and A260/230 ratios in the NanoDrop software (Thermo Scientific, Pittsburgh, PA, USA). For quantitative real-time RT-PCR analysis, first strand cDNA was synthesized from 500 ng total RNA using a PrimeScript Kit (TaKaRa, Dalian, China) following the manufacturer’s protocol. Real-time RT-PCR was carried out using SYBR Premix Ex Taq II (TaKaRa, Dalian, China), and analysis was performed on a 20 μL mixture containing 10 μL SYBR Premix Ex Taq II, 2 μL diluted cDNA and 0.8 μL of forward and reverse primers. The amplification was carried out as follows: 95 °C for 1 min, followed by 45 cycles at 95 °C for 10 s, 52 °C for 30 s, and 72 °C for 30 s. The gene encoding the ubiquitin-conjugating protein UBI-3 (GenBank accession no. AY486137.1) was amplified from pepper plants as a reference gene for normalization of CaF-box cDNA samples [46]. The primer sequences used for real-time RT-PCR are shown in Table 1. On the other hand, total RNA of CaF-box silence and control plant were used to examine the expression of three cold stress-related genes (*ERD15*, *RD22*, and *KIN1*). The corresponding specific primers were listed in Table 1. Relative expression levels were determined using the comparative threshold method (2^−ΔΔ^*^C^*^t^) [47]. All samples were performed in triplicate and each included at least three independent biological replicates.

### 3.6. Virus-Induced Gene Silencing of *CaF-Box* in Pepper

The pTRV2:*CaF-box* construct was engineered to include a 330 bp fragment of *CaF-box* cloned from a pepper cDNA template using gene-specific forward (5′-CTTGGTTGTGATTTTCTTGG-3′) and reverse (5′-CGCGGATCCCGCTGCACCCCTTTCAC-3′) primers containing a *Bam*H I restriction site (underlined). The resulting PCR product was cloned into vector pMD19T (Takara, Dalian, China), the resultant construct was digested with *Xba* I and *Bam*H I, and the CaF-box fragment was inserted into the *Xba* I-*Bam*H I site of pTRV2 to form pTRV2-*CaF-box. Agrobacterium tumefaciens* GV3101 containing either pTRV1 or pTRV2-*CaF-box* were injected into pepper and plants were grown as described by Wang *et al.* (2013) [38]. 49 plants were used for the silencing assay.

### 3.7. TBARS and Electrolyte Leakage Level Assay

The amount of lipid peroxidation in the chloroplast membranes was estimated by measuring TBARS produced by the thiobarbituric acid reaction as described previously by Dhindsa *et al.* (1981) [48], with modifications. The crude extract was mixed with the same volume of a 0.5% (*w*/*v*) thiobarbituric acid solution containing 5% (*w*/*v*) trichloroacetic acid. The mixture was heated at 100 °C for 15 min, cooled quickly, and centrifuged at 10,000 rpm for 10 min. The supernatant was used to measure the absorbance at 532, 600, and 450 nm. The TBARS concentration was calculated according to the following formula: (TBARS) = 6.45 × (A532 − A600) − (0.56 × A450).

To assess membrane permeability, electrolyte leakage was measured according to the method described by Dionisio-Sese and Tobita (1998) [49]. Briefly, 10 leaf disks (1 cm in diameter) were collected from the upper, fully expanded youngest leaves of two randomly chosen plants per replicate and washed with distilled water to remove surface contamination. The disks were placed individually in test tubes containing 10 mL distilled water, and incubated in a water bath at room temperature for 2 h. The initial electrical conductivity (EC1) of the medium was measured using a DDS-307 electrical conductivity analyzer (Shanghai Precision Scientific Instrument Co., Ltd., Shanghai, China). The samples were autoclaved at 100 °C for 30 min to release all of the electrolytes before cooling to 25 °C to obtain the final electrical conductivity (EC2). Electrolyte leakage was calculated as EC1/EC2 and expressed as a percentage.

### 3.8. Statistical Analysis

All data were expressed as the mean ± SD of three independent replicates (*n* = 3). Data from replicates of the three experiments were pulled together for one-way analysis of Variance (ANOVA), and differences in the mean values of different treatments were determined using the least significant difference (LSD) method. Statistical procedures were performed using the Statistical Analysis System software (SAS Institute, version 8.2, Cary, NC, USA). Values of *p* ≤ 0.05 were considered statistically significant.

## 4. Conclusions

In conclusion, we have isolated a novel pepper *F-box* protein-encoding gene, *CaF-box*, from the leaves of pepper cultivar P70. *CaF-box* transcript levels exhibited tissue differences, and the expression patterns of *CaF-box* were examined in response to abiotic stress and plant hormone treatment. *CaF-box* was induced strongly under cold stress treatment, and significantly enhanced during the early stages of NaCl stress, before mRNA levels decreased sharply to below those of control plants. In contrast, expression of *CaF-box* was downregulated by osmotic and heavy metal stress. Moreover, *CaF-box* expression was significantly upregulated by ABA and SA treatment. Virus-induced gene silencing (VIGS) revealed that mutant plants lacking *CaF-box* were more susceptible to low temperature treatment than control plants. Taken together, these results suggest that *CaF-box* plays an important role in the defense response to abiotic and biotic stress resistance. In the future we will perform genetic transformation studies in order to further explore the functional role of *CaF-box* in pepper.

## Figures and Tables

**Table 1.** Primer sequences used for Real-time RT-PCR analysis.

Gene	Primer	Sequence (5′-3′)
*CaUbi*3	Q*CaUbi*3-F	TGTCCATCTGCTCTCTGTTG
	Q*CaUbi*3-R	CACCCCAAGCACAATAAGAC
*CaF-box*	Q*CaF-box*-F	CAAGGTTCAATGTGTGTTACC
	Q*CaF-box*-R	ATGATGATACAAATACAGTGCC
*ERD15*	Q*ERD15*-F	CCAGCGAAATGGGGAAAC
	Q*ERD15*-R	ACAAAGGTACAGTGGTGGC
*RD22*	Q*RD22*-F	GCGTTGGCAGCGGAAAA
	Q*RD22*-R	GCGTTAGGATCGTCGTGG
*KIN1*	Q*KIN1*-F	AAATGTCAGAGACCAACAAGAA
	Q*KIN1*-R	CTACTTGTTCAGGCCGGTCTT

F: forward primer; R: reverse primer.
